# Evaluation of Individual and Combined Applications of Serum Biomarkers for Diagnosis of Hepatocellular Carcinoma: A Meta-Analysis

**DOI:** 10.3390/ijms141223559

**Published:** 2013-12-02

**Authors:** Bin Hu, Xiaohui Tian, Jie Sun, Xiangjun Meng

**Affiliations:** 1 Department of Gastroenterology, Shanghai First People’s Hospital, School of Medicine, Shanghai Jiaotong University, 100 Haining Road, Shanghai 200080, China; E-Mails: hubinsjtu@163.com (B.H.); tianxiaohui01@163.com (X.T.); Sunjie0426@126.com (J.S.); 2 Department of Gastroenterology, Shanghai Sixth People’s Hospital, School of Medicine, Shanghai Jiaotong University, 600 Yishan Road, Shanghai 200233, China

**Keywords:** hepatocellular carcinoma, biomarkers, detection, diagnosis

## Abstract

The clinical value of Serum alpha-fetoprotein (AFP) to detect early hepatocellular carcinoma (HCC) has been questioned due to its low sensitivity and specificity found in recent years. Other than AFP, several new serum biomarkers including the circulating AFP isoform AFP-L3, des-gamma-carboxy prothrombin (DCP) and Golgi protein-73 (GP73) have been identified as useful HCC markers. In this investigation, we review the current knowledge about these HCC-related biomarkers, and sum up the results of our meta-analysis on studies that have addressed the utility of these biomarkers in early detection and prognostic prediction of HCC. A systematic search in PubMed, Web of Science, and the Cochrane Library was performed for articles published in English from 1999 to 2012, focusing on serum biomarkers for HCC detection. Data on sensitivity and specificity of tests were extracted from 40 articles that met the inclusion criteria, and the summary receiver operating characteristic curve (sROC) was obtained. A meta-analysis was carried out in which the area under the curve (AUC) for each biomarker or biomarker combinations (AFP, DCP, GP73, AFP-L3, AFP + DCP, AFP + AFP-L3, and AFP + GP73) was used to compare the diagnostic accuracy of different biomarker tests. The AUC of AFP, DCP, GP73, AFP-L3, AFP + DCP, AFP + AFP-L3, and AFP + GP73 are 0.835, 0.797, 0.914, 0.710, 0.874, 0.748, and 0.932 respectively. A combination of AFP + GP73 is superior to AFP in detecting HCC and differentiating HCC patients from non-HCC patients, and may prove to be a useful marker in the diagnosis and screening of HCC. In addition, the AUC of GP73, AFP + DCP and AFP + GP73 are better than that of AFP. The clinical value of GP73, AFP + DCP, or AFP + GP73 as serological markers for HCC diagnosis needs to be addressed further in future studies.

## 1. Introduction

Hepatocellular carcinoma (HCC) is the third leading cause of cancer-related death worldwide. The incidence and mortality rates of HCC are almost equal because most HCC patients are diagnosed at an advanced stage. Therefore, the prognosis of HCC patients is generally poor, with a five-year survival rate lower than 5%. Alpha-fetoprotein (AFP) is the most commonly used serological biomarker in clinical practice. AFP, along with hepatic ultrasonography, is employed in the detection of HCC in high-risk patients with cirrhosis [1]. However, the clinical diagnostic accuracy of AFP is unsatisfactory due to low sensitivity and specificity. Therefore, there is an urgent need for developing better HCC-specific biomarkers [2,3]. Recent studies have identified other potential biomarkers for early detection of HCC, including the circulating AFP isoform AFP-L3, des-gamma-carboxy prothrombin (DCP), and Golgi protein-73 (GP73), although these biomarkers have been used in the clinic [4–6], the practical value of these markers has yet to be fully evaluated. So, it is meaningful to assess the value of these markers individually or for combined application in the clinic.

## 2. Results and Discussion

### 2.1. Results

**Figure 1.**
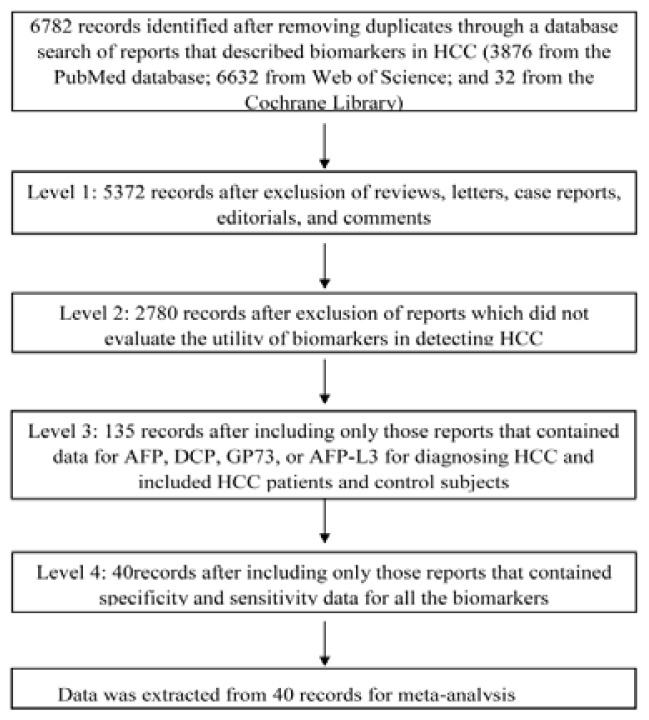
Literature screening was performed at four levels. Level 1, reviews, letters, case reports, editorials, and comments were excluded from the papers identified using the above search strategy. Level 2, articles in which biomarkers were not evaluated for their utility in detecting hepatocellular carcinoma (HCC) were excluded. The full texts of reports that met the above criteria were obtained with duplicate articles excluded. Level 3, the content of the articles was analyzed to ensure that the serum biomarkers in the study included Alpha-fetoprotein (AFP), des-gamma-carboxy prothrombin (DCP), Golgi protein-73 (GP73), or circulating AFP isoform AFP-L3, and that these biomarkers were used just for diagnosing HCC. The data pertaining to other biomarkers were excluded from further analysis. Articles were further screened to ensure that the studies included data pertaining to patients with HCC and appropriate control populations. At Level 4, only reports with sensitivity and specificity data for the biomarkers were selected. A total of 40 reports met the inclusion criteria and were selected for meta-analysis.

A total of 40 studies [7] were included in the meta-analysis, 35 for AFP (biomarker **1**), 15 for DCP (biomarker **2**), nine for GP73 (biomarker **3**), 15 for AFP-L3 (biomarker **4**), eight for AFP+DCP (biomarker **5**), three for AFP+AFP-L3 (biomarker **6**), and two for AFP+GP73 (biomarker **7**). The literature search strategy is depicted below (Table 1). Literature screening was performed at four levels (Figure 1). We extracted data from the selected papers on authors, country, year of publication, journal, number of patients, test methods and results, sensitivity, specificity, and cut-off points for the biomarkers (Table 2).

**Figure 2.**
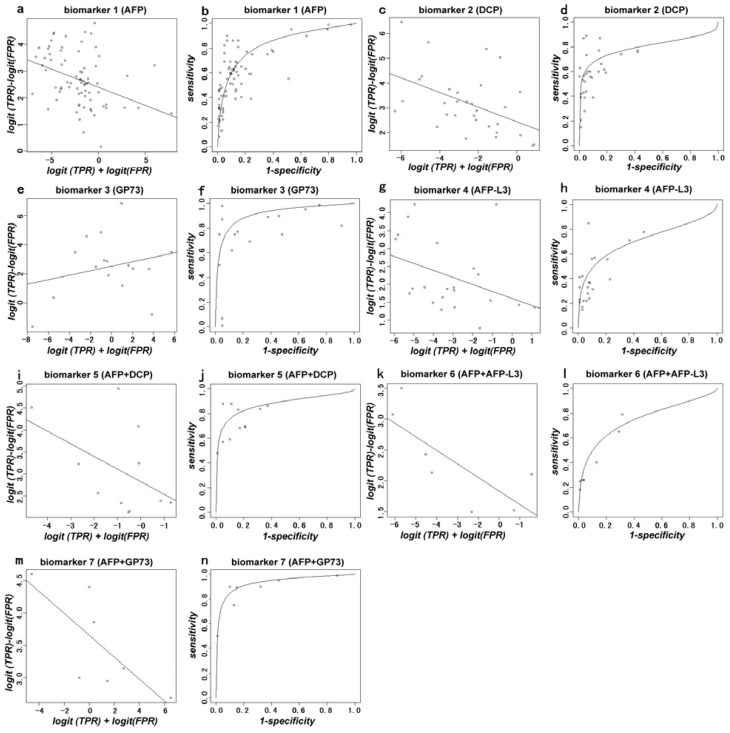
Plots for calculating the area under the curve (AUC) and *D* (log odds ratio) against *S* (implicit threshold) for biomarker **1** to biomarker **7**. The *S*-value represents the positive rate of the biomarker for detecting HCC. *S* = logit (TPR) + logit (FPR), where TPR is the true positive rate (sensitivity) and FPR is the false positive rate (1-specificity) for AFP. *D* represents the ability of distinguishing HCC and the control. *D* = logit (TPR) − logit (FPR) (**a**, **c**, **e**, **g**, **i**, **k**, **m**). The AUC of biomarkers **1** to biomarker **7** are 0.835, 0.797, 0.914, 0.710, 0.874, 0.748, 0.932 respectively (**b**, **d**, **f**, **h**, **j**, **l**, **n**).

In this meta-analysis, AFP was considered to be the reference biomarker. The area under the curve (AUC) and *S*-values were the major indicators. The AUC for **1**, **2**, **3**, **4**, **5**, **6** and **7** biomarkers or the associated compounds were 0.835, 0.797, 0.914, 0.710, 0.874, 0.748, and 0.932 respectively. It was found that the AUC of biomarkers **3**, **5** and **7** were superior to that of the reference biomarker **1**, while the AUC of biomarkers **2**, **4** and **6** were inferior to that of the reference biomarker **1** (Table 3). The *S-*value represents the positive rate of the biomarkers for detecting HCC. Using biomarker **1** as a reference marker, the *S*-values of biomarkers **2**, **3**, **4**, **5** and **6** were not significantly different from that of AFP (*p* > 0.05). The *S*-value of biomarker **7** was significantly different as compared with AFP (*p <* 0.05) (Table 4). The plots for calculating the pooled AUC, and the *S*- and *D*-values for each biomarker are shown in (Figure 2).

### 2.2. Discussion

HCC remains one of the most common malignant tumors. Early diagnosis and early surgical extraction are imperative for improving the survival of HCC patients [1,4,5]. AFP, a specific glycoprotein produced primarily by the fetal liver has been the most practical and widely used serum biomarker for HCC diagnosis. However, its sensitivity and specificity varied significantly ranging from 40%–65% and 76%–96%, respectively [45,48,49]. The AFP value is often elevated at a milder level in patients with chronic hepatitis C infection in the absence of HCC [27]. An AFP value >400 ng/mL is considered ideal for diagnosing HCC [27]. Even though normal AFP levels can be seen in approximately one-third of patients with HCC [49], a large number of HCC patients have AFP values <400 ng/mL, making them very difficult to undergo detection and prognosis of HCC [49]. Other conventional clinical tumor associated biomarkers such as CEA, CA199, CA724 *etc.* are not practical in the clinic because of their poor sensitivity and specificity. The present situation requires an urgent need to explore new markers to overcome these drawbacks in liver cancer diagnosis.

An ideal serum biomarker should be both sensitive and specific for HCC detection at an early stage, and be easy to test, reproduce, as well as be non-invasive [50]. With the latest developments in molecular techniques, several new HCC-specific biomarkers including AFP-L3, DCP and GP73 have been discovered [51,52]. These new markers have been investigated for their diagnostic accuracy and potential for HCC detection [53,54]. However, the clinical usefulness of these biomarkers needs to be carefully evaluated and validated. Thus, we aimed to evaluate the utility of the biomarkers individually, as well as their combined application in the early detection of HCC and for their usefulness in therapeutic decision-making.

AFP-L3, one of the AFP isoforms, has a high binding affinity to lectin *Lens culinaris* agglutinin. It has been reported that AFP-L3 is a more valuable index than total AFP for early diagnosis of HCC [24,51]. The proportion of AFP-L3 over the total AFP concentration has been used as a marker for early diagnosis and assessment of the therapeutic effect as well as prognosis of HCC [51]. AFP-L3 was found to be associated with liver dysfunction, poor differentiation, and other biologically malignant characteristics [48]. If total AFP concentration is below 10 ng/mL, the absolute value of AFP-L3 would be hard to be detected. However, AFP-L3 instead of AFP can be detected in the serum of some patients with tumors smaller than two centimeters in size. Generally, AFP-L3 has been detected in approximately one-third of HCC patients with cutoff values of 10%–15% (percentage of AFP-L3 over AFP) [2,55]. Therefore, percentage of AFP-L3 is often used when AFP concentration is above 10 ng/mL, especially within levels between 10–200 ng/mL [8]. In the clinic, it is a diagnostic dilemma for patients with total AFP values of 10–200 ng/mL [8]. For these cases, AFP-L3 may be a better complement index for diagnosing HCC when combined with AFP. However, because its sensitivity and specificity range from 36%–96%, and 89%–94%, respectively [34,48,49,53,56,57], drawing a conclusion requires caution. For example, Nouso K *et al*. found that the sensitivity of AFP-L3 in patients of HCC with low AFP (under 20 ng/mL) was 51.5%, 13.3%, and 8.7% at cutoff levels of 5%, 10%, and 15%, respectively [58]. Leerapun, A. *et al*. found that in patients with total AFP values of 10–200 ng/mL, the AFP-L3 was greater than 10% and had a sensitivity of 71% and a specificity of 63% for diagnosing HCC [8]. With an AFP-L3 greater than 35%, the specificity for HCC diagnosis reached 100% while reducing sensitivity to 33% [8]. Nonetheless, the combination of AFP-L3% and AFP significantly improved the specificity (100%) of diagnosis of HCC [8]. Thus, they proposed that the AFP-L3% should be taken as a potential marker for AFP in the diagnosis of HCC, although no prognostic significance for AFP-L3% was observed after adjustment for total AFP [8]. Our meta-analysis result showed that AFP-L3 alone or combined with AFP was not superior to AFP (AUC: 0.710, 0.748 *vs*. 0.835) (Table 3). However, significant variations for interpreting the present studies arose due to different assay methods, cutoff values, and study populations.

As a new serum biomarker in patients with HCC, DCP, or prothrombin, induced by vitamin K absence-II (PIVKA-II) was first described by Liebman *et al*. [59]. DCP is an abnormal protein that is increased in the serum of patients with HCC. With a cutoff value of 40 mAU/mL, the sensitivity and specificity of DCP for diagnosing HCC ranges from 28%–89%, and 87%–96% respectively [19,25,33,60–65]. DCP at a 125 mAU/mL had a high sensitivity (89%) and specificity (95%) for differential diagnosis of HCC from cirrhosis and chronic hepatitis [25]. The areas under the ROC curve for DCP (125 mAU/mL) and AFP (11 ng/mL) were 0.928 *vs*. 0.810, respectively [25]. Another case-controlled study comparing the AFP value of diagnosing HCC with DCP showed that a cutoff value of 60 mAU/mL for DCP had a higher sensitivity than AFP (with a cutoff value 20 ng/mL) (75.5% *vs*. 68.4%) [60]. Therefore, DCP is considered as a valuable complement prognostic predictor for HCC [66,67]. Nevertheless, just as our meta-analysis showed, DCP was not statistically better than AFP (AUC: 0.797 *vs*. 0.835), albeit combined measurement of DCP and AFP was superior to AFP alone (AUC: 0.874 *vs*. 0.835) (Table 3). As a biomarker of HCC, more appropriate and accurate evaluations of DCP are expected in future studies.

GP73, also called Golgi phosphoprotein 2 (GOLPH2), is a resident Golgi transmembrane glycoprotein with 400 amino acids and the 73kDa molecular weight was found up-regulated in expression in virus-infected hepatocyte [52]. Several recent studies indicate that GP73 is one of the most promising serum markers for HCC. Although there are studies reporting that the sensitivity of GP73 was higher than that of AFP in the diagnosis of early HCC (62% *vs*. 25%), the potential clinical value of GP73 as a better serum biomarker than AFP remains controversial [7,17,31,54,68]. A large cohort study of HCC by Mao *et al*. showed that GP73 was a valuable tumor biomarker for HCC and was superior to AFP with respect to sensitivity and specificity after comparing the adjusted factors such as the likelihood ratio and predictive value, which are independent of the age and gender of the subjects [23]. They also found that combined measurement of GP73 and AFP could further increase the sensitivity for the detection of HCC. Using 8.5 relative units as the cut-off value, the sensitivity and specificity of serum GP73 for HCC were 74.6% (95% confidence interval (CI), 71.5%–77.6%) and 97.4% (95% CI, 96.8%–98.3%) [23]. Combined measurement of GP73 and AFP increased the sensitivity for HCC to 89.2% (95% CI, 86.7%–91.5%), with a specificity of 85.2% (95% CI, 83.4%–86.4%) [23], which indicated that serum GP73 was dramatically elevated in patients with HCC and that the sensitivity and specificity of GP73 for HCC might be superior to that of AFP [23]. Another meta-analysis by Zhou *et al*. found that, as an independent marker for diagnosis of HCC, GP73 is comparable to AFP [69]. In our study, the result showed that GP73 and AFP combined were dramatically superior to AFP (AUC: 0.914, 0.932 *vs*. 0.835), which was similar to the results above (Table 3). However, there were several drawbacks in our study. For instance, the data extracted from the paper was not enough to conclude for certain that GP73 and AFP were more effective when applied together. Moreover, the assay methods and the cutoff values varied in these studies.

The results of this meta-analysis showed that the AUC of biomarkers **3**, **5** and **7** were superior to that of the reference biomarker AFP, while the AUC of biomarkers **2**, **4**, and **6** were inferior to that of the reference biomarker AFP. Based on our tendency to conclude that GP73, together with DCP + AFP, with the combined measurement of GP73 + AFP could increase detection of HCC, this may be superior to AFP for diagnosis and screening in the early stages of HCC. Using biomarker **1** (AFP) as the reference marker, there is no significant difference in the *S*-value between biomarkers **2**, **3**, **4**, **5** and **6** (*p* > 0.05), while the *S*-value of biomarker **7** (AFP + GP73) was significantly different (*p* < 0.05), suggesting that biomarker **7** may be more valuable and useful in clinical practice, although the compound value of AFP + GP73 still calls for further research and evaluation.

The clinical value of a biomarker depends not only on the high sensitivity and specificity but also on the universality and availability for practice. Technologies of testing assays for these biomarkers vary, including: ELISA, LiBASys, μTAS, IAUEC, ECLIA, EIA, LAEC, and immunoblot. Some may not been used worldwide and their costs differ significantly. However, with the optimization and improvement of technology, such problems are expected to be resolved. The costs of these technologies could not be extracted from the original citation papers, which suggests that in future studies, financial factors should be taken into consideration. According to the inclusion criteria, although the obtained reports of each biomarker may bring potential bias, they do not affect statistical analysis with Empower Stats software package (Empower Stats, X & Y Solutions, Boston, MA, USA). Therefore, more caution needs to be taken with the results.

## 3. Experimental Section

### 3.1. Identification of Studies

To assess biomarkers for HCC, a comprehensive literature search of original research articles published between January 1999 and July 2012 (cut-off date 1 July) was conducted using the PubMed database (http://www.ncbi.nlm.nih.gov/pubmed), Web of Science (http://www.isiknowledge.com), and Cochrane Library (http://www.thecochranelibrary.com).

### 3.2. Literature Screening

Literature screening was performed at four levels. At level 1, we excluded reviews, letters, case reports, editorials, and comments. At level 2, we excluded the articles in which biomarkers were not evaluated for their utility in detecting HCC and content duplicated articles. At level 3, we ensured the articles included serum biomarkers: AFP, DCP, GP73, or AFP-L3 for diagnosing HCC in their studies. The data pertaining to other biomarkers were excluded from further analysis. Articles were further screened to ensure that the data in those studies pertained to patients with HCC and appropriate control populations. At level 4, only reports with sensitivity and specificity data for the biomarkers were selected. A total of 40 reports met the inclusion criteria for meta-analysis.

### 3.3. Data Extraction

Data extracted from the selected papers included authors, country, year of publication, journal, number of patients, test methods and results, sensitivity, specificity, and cut-off points for the biomarkers.

### 3.4. Statistical Analysis

Data from disparate reports were summarized by the method described by Littenberg and Moses using a logistic transformation and linear regression we generated a summary receiver operating characteristic (sROC) curve [70]. The ROC curve has been demonstrated to be useful for summarizing the diagnostic accuracy of multiple reports, comparing technologies, detecting outliers, and identifying the optimum operating point of the test.

The sensitivity and specificity of the biomarkers from the included studies were logistically transformed, and then a linear regression line was fitted through the data points. This line was back-transformed to obtain the sROC curve [7], which is a compact description of the accuracy of the diagnostic test in many populations [48,70]. We did not extrapolate the curve beyond the range of the logistically transformed data.

The AUC, as one of the serum biomarkers, was obtained from the included studies. For each biomarker, the pooled AUC was calculated with the inverse standard errors as weights. The pooled AUC, together with similarly pooled standard errors, was used to compare the accuracy of these diagnostic tests [7].

Two parameters were used to describe the meta-analysis data S, a sum of the two transforms, is related to the frequency when the test is positive, which depends on the test threshold D, the difference between the two transforms, is a measure of the successful degree of the test to discriminate between the populations of healthy subjects and HCC [70].

AFP was taken as the reference marker for performing comparisons with other biomarkers. Student’s *t*-test was used to compare the pooled AUC of AFP and the pooled AUC of each new biomarker. Statistical analysis was performed using the Empower Stats software package (Empower Stats, X & Y Solutions, Boston, MA, USA). Differences were considered statistically significant at a *p <* 0.05.

## 4. Conclusions

The results of our meta-analysis suggested that AFP+GP73 is superior to AFP alone in diagnosing HCC and differentiating HCC patients from non-HCC patients, therefore, it might be a useful compound marker in the diagnosis and screening of potential HCC patients. In addition, the values of GP73, AFP + DCP and AFP + GP73 are also better than that of AFP alone. The clinical value of GP73, AFP + DCP, or AFP + GP73 as serological markers for early diagnosis of HCC needs to be evaluated further in future studies using stricter criteria.

## Figures and Tables

**Table 1.** Literature search strategy.

Database	Search criteria	Filters/Limits
PubMed	Search ((hepatocellular carcinoma [MeSH Terms]) and biological markers [MeSH Terms]) and (“1999” [Date—Publication]: “2012” [Date—Publication])	Humans; English
Web of Science	(hepatoma * or liver cell neoplasm * or hepatocellular neoplasm * or liver cell cancer * or hepatocellular cancer * or liver cell tumo * or hepatocellular tumo * or liver cell carcinom * or hepatocellular carcinom *) and (Biomarker * or Biological Marker * or Biologic Marker * or Biochemical Marker * or Serum Marker * or Clinical Marker *)	Publication date: 1999–present and language: English
Cochrane Library	#1 MeSH descriptor: [Carcinoma, Hepatocellular] explode all trees #2 MeSH descriptor: [Biological Markers] explode all trees	Dates: from 1999–2012

* A comprehensive literature search of original research articles published between January 1999 and July 2012 (cut-off date 1 July) assessing biomarkers for hepatocellular carcinoma (HCC) was conducted using the PubMed database, Web of Science, and Cochrane Library.

**Table 2.** Data from the selected papers.

Study	Country/District	Publication Year	Number	Biomarker	Test Methods	Sensitivity	Specificity	Cutoff	Journal
Leerapun [8]	USA	2007	52	AFP-L3	LiBASys	0.710	0.630	10%	*Clin. Gastroenterol. Hepatol.*
						0.330	1.000	35%	

Cedrone [9]	Italy	2000	74	AFP	ELISA	0.200	0.990	200 ug/L	*Hepato Gastroenterol.*
						0.550	0.880	20 ug/L	

Wang [10]	Taiwan, China	2005	61	AFP	ELISA	0.590	0.770	20 ng/mL	*World J. Gastroenterol.*
				DCP	EIA	0.770	0.860	40 mAU/mL	
				AFP + DCP	ELISA + EIA	0.836	0.682	20 ng/mL/40 mAU/mL	

Durazo [11]	USA	2008	144	AFP	IAUEC	0.690	0.870	25 ng/mL	*J. Gastroenterol. Hepatol.*
				DCP	ELISA	0.870	0.850	84 mAU/mL	
				AFP-L3	LAEC	0.560	0.900	10%	

Gianluigi [12]	Italy	2007	499	AFP	ELISA	0.410	0.940	18.8 ng/mL/20.5 IU/mL	*Clin. Chim. Acta*

Giannelli [13]	Italy	2005	120	AFP	ELISA	0.450	0.870	12.6 ng/mL/13.7 IU/mL	*Int. J. Cancer*

Toyoda [14]	Japan	2011	270	AFP-L3	μTAS	0.394	0.770	7%	*Cancer Sci.*

Okuda [15]	Japan	1999	60	DCP	EIA	0.617	0.821	30 mAU/mL	*Cancer*
						0.600	0.863	35 mAU/mL	
						0.600	0.923	40 mAU/mL	
						0.583	0.949	45 mAU/mL	
						0.567	0.966	50 mAU/mL	
						0.567	0.966	55 mAU/mL	
						0.533	0.974	60 mAU/mL	

Hsia [16]	Taiwan, China	2007	26	AFP	ELISA	0.620	0.880	20 ng/mL	*Eur. J. Surg. Oncol.*

Hu [17]	China	2010	31	AFP	ELISA	0.480	0.970	36 ug/L	*Med. Oncol.*
				AFP + GP73	ELISA/western blotting	0.770	0.840	7.4 RU	

Hussein [18]	Egypt	2008	49	AFP	ELISA	0.900	0.930	7.7 ng/mL	*Indian J. Cancer*

Ikoma [19]	Japan	2002	63	AFP	ELISA	0.510	0.830	20 ng/mL	*Hepato Gastroenterol.*
				DCP	ELISA	0.390	0.960	16 mAU/mL	
				AFP + DCP	ELISA/ELISA	0.830	0.840	20/16	

Ertle [20]	Germany	2011	170	AFP	ELISA	0.310	0.960	200 ng/mL	*J. Hepatol.*
				DCP	LiBASys	0.600	0.940	7.5 ng/mL	
				AFP-L3	LiBASys	0.410	0.990	10%	

Yamamoto [21]	Japan	2009	714	AFP	immunometric assay	0.649	0.829	11 ng/mL	*Ann. Surg. Oncol.*
						0.608	0.861	13 ng/mL	
						0.513	0.908	20 ng/mL	
						0.304	0.986	100 ng/mL	
						0.247	0.990	200 ng/mL	
				DCP	two-step enzyme immunoassay	0.734	0.947	20 mAU/mL	
						0.628	0.994	30 mAU/mL	
						0.559	0.998	40 mAU/mL	
						0.419	1.000	100 mAU/mL	
						0.391	1.000	125 mAU/mL	

Volk [22]	USA	2007	84	AFP	IAUEC	0.860	0.930	150 mAU/mL	*Cancer Biomark.*
						0.690	0.910	23 ng/mL	
				AFP-L3	IAUEC	0.570	0.880	3%	
				AFP + DCP	IAUEC/ELISA	0.880	0.890	23 ng/mL/150 mAU/mL	

Mao [23]	China	2010	789	AFP + GP73	ELISA/Immunoblot	0.892	0.852	35 ng/mL/8.5 RU	*Gut*
				GP73	Immunoblot	0.750	0.970	8.5 RU	
				AFP	ELISA/Immunoblot	0.580	0.850	35 ug/L	

Yamamoto [24]	Japan	2010	96	AFP	ELISA	0.400	0.830	15 ng/mL	*J. Gastroenterol.*
						0.390	0.870	20 ng/mL	
						0.280	0.960	124 ng/mL	
						0.220	0.960	200 ng/mL	
				DCP	Immunoblot	0.770	0.580	20 mAU/mL	
						0.590	0.810	30 mAU/mL	
						0.550	0.910	40 mAU/mL	
						0.520	0.960	60 mAU/mL	
				AFP-L3	IAUEC	0.240	0.920	5%	
						0.220	0.960	10%	
						0.170	0.970	15%	
						0.150	0.970	20%	
				AFP + DCP	Immunoblot	0.690	0.790	20 ng/mL/40 mAU/mL	
						0.680	0.830	20 ng/mL/60 mAU/mL	
						0.590	0.900	400 ng/mL/40 mAU/mL	
						0.570	0.950	400 ng/mL/60 mAU/mL	
				AFP + AFP-L3	Immunoblot/IAUEC	0.400	0.870	20 ng/mL/irrespective	
						0.260	0.960	20–400 ng/mL/10%	
						0.260	0.970	20–400 ng/mL/15%	
						0.180	0.990	400 ng/mL/irrespective	

Marrero [25]	USA	2003	55	AFP	IAUEC	0.770	0.790	11 ng/mL	*Hepatology*
						0.680	0.860	20 ng/mL	
						0.470	0.980	100 ng/mL	
						0.340	1.000	400 ng/mL	
				DCP	ELISA	0.890	0.950	125 mAU/mL	
						0.870	0.970	150 mAU/mL	
				AFP + DCP	IAUEC/ELISA	0.880	0.950	logAFP + 4.6 * logDCP	

Colombo [26]	Italy	2001	55	AFP	ELISA	0.390	0.760	20 ng/ml	*Clin. Liver Dis.*
						0.130	0.970	100 ng/mL	

Nguyen [27]	USA	2002	163	AFP	ELISA	0.784	0.611	10 ng/mL	*Hepatology*
						0.630	0.799	20 ng/mL	
						0.506	0.893	50 ng/mL	
						0.414	0.973	100 ng/mL	
						0.321	1.000	200 ng/mL	

Marrero [28]	USA	2009	419	AFP	LiBASys	0.590	0.900	20 ng/mL	*Gastroenterology*
				DCP	ELISA	0.740	0.700	150 mAU/mL	
				AFP-L3	LiBASys	0.420	0.970	10%	
				AFP + DCP	ELISA + LiBASys	0.860	0.630	20 ng/mL/150 mAU/mL	

Shafie [29]	Egypt	2012	31	AFP	ELISA	0.810	0.850	32.64 ng/mL	*Life Sci. J.*
						0.770	0.600	28.51 ng/mL	
				AFP + GP73	ELISA	0.870	0.950	32.64 ng/mL/7.62 ng/mL	
						0.900	0.900	28.51 ng/mL/7.62 ng/mL	

Morota [30]	Japan	2011	UN	AFP	ELISA	0.630	0.920	15.3 ug/L	*Clin. Chem. Lab. Med.*
				AFP + GP73	ELISA	0.890	0.620	94.7 ug/L	

Marrero [31]	USA	2005	144	AFP	ELISA	0.300	0.960	99 ug/L	*J. Hepatol.*
				AFP + GP73	ELISA/Immunoblot	0.690	0.750	10.0 RU	

Tong [32]	USA	2001	31	AFP	ELISA	0.410	0.950	24 ng/mL	*J. Gastroenterol. Hepatol.*
						0.410	0.940	21 ng/mL	
						0.450	0.940	19 ng/mL	
						0.550	0.930	16 ng/mL	
						0.590	0.910	13 ng/mL	
						0.860	0.890	11 ng/mL	
						0.860	0.850	8 ng/mL	

Nomura [33]	Japan	1999	36	AFP	ELISA	0.590	0.760	20 ng/mL	*Am. J. Gastroenterol.*
				DCP	Immunoblot	0.280	0.960	40 mAU/mL	
				AFP-L3	ECLIA	0.220	0.940	10%	

Oka [34]	Japan	2001	388	AFP	ELISA	0.550	0.490	20 ng/mL	*J. Gastroenterol. Hepatol.*
				AFP-L3	LAEC	0.280	0.930	10%	
						0.210	0.990	15%	

Ozkan [35]	Turkey	2011	75	AFP	ELISA	0.820	0.950	4.36 ug/L	*Digestion*
						0.690	0.950	13 ng/mL	
						0.760	0.950	8.46 ng/mL	
						0.600	0.980	20 ng/mL	
						0.460	1.000	100 ng/mL	
						0.390	1.000	200 ng/mL	
				AFP + GP73	ELISA	0.070	0.950	20 ng/mL/2.36 ug/L	
						0.820	0.090	20 ng/mL/0.078 ng/mL	
						0.000	0.950	20 ng/mL/24.43 ng/mL	

Peng [36]	Taiwan, China	1999	205	AFP	ELISA	0.450	1.000	200 ug/L	*Hepato Gastroenterol.*
						0.650	0.870	20 ug/L	

Porta [37]	Italy	2008	30	AFP	ELISA	0.630	0.880	12.8 ng/mL	*Ann. Oncol.*

Romeo [38]	Italy	2010	86	AFP	μ TAS	0.465	0.868	20 ng/mL	*J. Clin. Oncol.*
				DCP	μ TAS	0.663	0.842	0.5 ng/mL	
						0.558	0.921	0.75 ng/mL	
						0.209	1.000	2.5 ng/mL	
						0.151	1.000	7.5 ng/mL	
				AFP-L3	μ TAS	0.779	0.526	5 ng/ml	
						0.558	0.789	7 ng/mL	
						0.314	0.895	10 ng/mL	
				AFP + DCP	μ TAS	0.698	0.789	20 ng/mL + 0.75 ng/mL	
				AFP + AFP-L3	μ TAS	0.651	0.711	20 ng/mL + 10 ng/mL	
						0.791	0.684	20 ng/mL + 1010 ng/mL	

Sassa [39]	Japan	1999	61	AFP	ELISA	0.080	1.000	200 ng/mL	*Eur. J. Gastroenterol. Hepatol.*
				DCP	immunoassay	0.440	0.950	40 mAU/mL	
				AFP-L3	LAEC	0.230	0.990	10%	
				AFP + DCP	ELISA/immunoassay	0.480	0.990	200ng/mL/40 mAU/mL	
				AFP + AFP-L3	ELISA/LAEC	0.250	0.990	200 ng/mL/10%	

Shi [40]	China	2011	73	AFP	ELISA	0.750	0.750	400 ug/L	*Technol. Cancer Res. Treat*
				AFP + GP73	ELISA	0.980	0.950	400 ug/L/100.0 ug/L	

Shimauch [41]	Japan	1999	21	AFP-L3	Immunoblot	0.330	0.930	10%	*Oncol. Rep.*
				DCP	Immunoblot	0.430	0.970	40mAU/mL	

Sterling [42]	USA	2009	74	AFP	LiBASys	0.990	0.200	200 ng/mL	*Clin. Gastroenterol. Hepatol.*
						0.608	0.711	20 ng/mL	
				DCP	LiBASys	0.760	0.580	40 mAU/mL	
					LiBASys	0.390	0.900	7.5 ng/mL	
				AFP+DCP	LiBASys	0.703	0.634	20 ng/mL/7.5 ng/mL	
				AFP + AFP-L3	LiBASys	0.689	0.664	20 ng/mL/10	
				AFP-L3	LiBASys	0.365	0.916	10%	

Sun [43]	China	2009	79	AFP-L3	ACSC method	0.848	0.925	10	*J. Gastroenterol. Hepatol.*

Tian [44]	China	2010	153	AFP	ELISA	0.950	0.470	13.6 ug/L	*Int. J. Cancer*
				AFP + GP73	ELISA	0.750	0.520	113.8 ug/L	

Trevisani [45]	Italy	2001	170	AFP	ELISA	0.600	0.906	20 ug/L	*J. Hepatol.*
						0.624	0.894	16 ug/L	
						0.312	0.988	100 ug/L	
						0.224	0.994	200 ug/L	
						0.171	0.994	400 ug/L	

Wang [46]	USA	2009	164	AFP	ELISA	0.950	0.210	NK	*Cancer Epidemiol. Biomark. Prev.*
						0.500	0.980	NK	
						0.750	0.640	NK	
						0.900	0.360	NK	
						0.950	0.210	NK	
						1.000	0.040	NK	

				GP73	Immunoblot	0.500	0.970	NK	
						0.750	0.860	NK	
						0.900	0.540	NK	
						0.950	0.350	NK	
						1.000	0.250	NK	

				AFP + GP73	ELISA/Immunoblot	0.500	0.990	NK	
						0.750	0.870	NK	
						0.900	0.680	NK	
						0.950	0.550	NK	
						1.000	0.130	NK	

LI [47]	China	2009	50	AFP	ELISA	0.250	0.970	11.23 ng/mL	*Hepatology*
				AFP + GP73	Immunoblot	0.620	0.880	14.37 RU	

AFP: alpha-fetoprotein; DCP: des-gamma-carboxy prothrombin; GP73: Golgi protein-73; AFP-L3: Alpha-fetoprotein L3 isoform, ELISA: enzyme-linked immunosorbent assay; ACSC: *Lens culinaris* agglutinin (LCA)-coupled spin column; EIA: conventional enzyme immunoassay; μTAS: micro-total analysis system; IAUEC: immunometric assays utilizing enhanced chemiluminescence; ECLIA: immunoassay using the electrochemiluminescence detection system; LiBASys: LiBASys automated immunologic analyzer; LAEC: lecithin-affinity electrophoresis coupled with antibody-affinity blotting; NK: not known.

* : Multiply.

**Table 3.** The area under the curve (AUC) for each marker in the meta-analysis.

Biomarker	Number of Studies	AUC
1 (AFP)	35	0.835
2 (DCP)	15	0.797
3 (GP73)	9	0.914
4 (AFP-L3)	15	0.710
5 (AFP+DCP)	8	0.874
6 (AFP+AFP-L3)	3	0.748
7 (AFP+GP73)	3	0.932

AFP: alpha-fetoprotein; DCP: des-gamma-carboxy (abnormal) prothrombin; GP73: golgi protein-73; AFP-L3: Alpha-fetoprotein L3 isoform.

**Table 4.** The *S*-values of the biomarkers in the meta-analysis.

	Estimate	Std. Error	*t* Value	*Pr* (>|t|)
(Intercept)	2.39257	0.15197	15.744	<2 × 10^−16^
S.Biomarker.**1**	0.13594	0.04532	2.999	0.00314
S.Biomarker.**2**	0.29219	0.10222	2.859	0.00483
S.Biomarker.**3**	0.15326	0.08245	1.859	0.06492
S.Biomarker.**4**	−0.19643	0.12226	−1.607	0.11012
S.Biomarker.**5**	−0.28662	0.20797	−1.378	0.17009
S.Biomarker.**6**	0.21913	0.17122	1.280	0.20250
S.Biomarker.**7**	−0.16597	0.13200	1.257	0.21050
Biomarker.**2**	0.08054	0.36171	0.223	0.82409
Biomarker.**3**	0.13195	0.31404	0.420	0.67494
Biomarker.**4**	−0.78803	0.47604	−1.655	0.09983
Biomarker.**5**	0.43157	0.40973	1.053	0.29381
Biomarker.**6**	0.77644	0.70680	1.099	0.27364
Biomarker.**7**	1.21688	0.48657	2.501	0.01341

The statistical significance level was set to *p <* 0.05. The *S*-value, which represents the positive rate of the biomarker for detecting HCC, was calculated as follows: *S* = logit (TPR) + logit (FPR), where TPR is the true positive rate (sensitivity) and FPR is the false positive rate (1-specificity).
